# Contribution of Efflux to the Emergence of Isoniazid and Multidrug Resistance in *Mycobacterium tuberculosis*

**DOI:** 10.1371/journal.pone.0034538

**Published:** 2012-04-06

**Authors:** Diana Machado, Isabel Couto, João Perdigão, Liliana Rodrigues, Isabel Portugal, Pedro Baptista, Bruno Veigas, Leonard Amaral, Miguel Viveiros

**Affiliations:** 1 Grupo de Micobactérias, Unidade de Microbiologia Médica, Instituto de Higiene e Medicina Tropical, Universidade Nova de Lisboa (IHMT/UNL), Lisboa, Portugal; 2 Centro de Recursos Microbiológicos (CREM), Faculdade de Ciências e Tecnologia, Universidade Nova de Lisboa, Caparica, Portugal; 3 Centro de Patogénese Molecular/URIA, Faculdade de Farmácia, Universidade de Lisboa, Lisboa, Portugal; 4 CIGMH, Departamento de Ciências da Vida, Faculdade de Ciências e Tecnologia, Universidade Nova de Lisboa, Caparica, Portugal; 5 COST ACTION BM0701 (ATENS), Brusells, Belgium; St. Petersburg Pasteur Institute, Russian Federation

## Abstract

Multidrug resistant (MDR) tuberculosis is caused by *Mycobacterium tuberculosis* resistant to isoniazid and rifampicin, the two most effective drugs used in tuberculosis therapy. Here, we investigated the mechanism by which resistance towards isoniazid develops and how overexpression of efflux pumps favors accumulation of mutations in isoniazid targets, thus establishing a MDR phenotype. The study was based on the *in vitro* induction of an isoniazid resistant phenotype by prolonged serial exposure of *M. tuberculosis* strains to the critical concentration of isoniazid employed for determination of drug susceptibility testing in clinical isolates. Results show that susceptible and rifampicin monoresistant strains exposed to this concentration become resistant to isoniazid after three weeks; and that resistance observed for the majority of these strains could be reduced by means of efflux pumps inhibitors. RT-qPCR assessment of efflux pump genes expression showed overexpression of all tested genes. Enhanced real-time efflux of ethidium bromide, a common efflux pump substrate, was also observed, showing a clear relation between overexpression of the genes and increased efflux pump function. Further exposure to isoniazid resulted in the selection and stabilization of spontaneous mutations and deletions in the *katG* gene along with sustained increased efflux activity. Together, results demonstrate the relevance of efflux pumps as one of the factors of isoniazid resistance in *M. tuberculosis*. These results support the hypothesis that activity of efflux pumps allows the maintenance of an isoniazid resistant population in a sub-optimally treated patient from which isoniazid genetically resistant mutants emerge. Therefore, the use of inhibitors of efflux should be considered in the development of new therapeutic strategies for preventing the emergence of MDR-TB during treatment.

## Introduction

Tuberculosis (TB) remains a serious public health threat around the world, and according to the World Health Organization, nearly two billion people are infected with *Mycobacterium tuberculosis*, with about 8.8 million of new TB cases and 1.3 million deaths in 2010 [1]. Moreover, multidrug resistant tuberculosis (MDR-TB), caused by *M. tuberculosis* simultaneously resistant to isoniazid and rifampicin, the two most effective anti-bacillary drugs used in TB therapy, represents a challenge to the control of the disease since 650,000 of the TB cases in 2010 are estimated to be MDR-TB cases [1].

Chromosomal gene mutation has been considered the single cause for antibiotic resistance in *M. tuberculosis*, with multidrug resistance arising as a consequence of sequential accumulation of spontaneous mutations in target genes [2]. Resistance to rifampicin is almost always due to point mutations in the *rpoB* gene encoding the β subunit of the RNA polymerase [3]. Furthermore, monoresistance to rifampicin is rare and almost all *M*. *tuberculosis* strains resistant to rifampicin are also resistant to isoniazid [2], [4], [5]. Isoniazid is a prodrug that requires activation by the catalase-peroxidase enzyme (KatG) [6] and its molecular target is InhA, a NADH-dependent enoyl acyl carrier protein reductase involved in the synthesis of mycolic acids [7]. The main mechanism of resistance to isoniazid is the occurrence of mutations in its activator, KatG [6], [8], whereas mutations in the *inhA* gene represent the second most common mechanism. Together, mutations in these two genes are responsible for approximately 75% of the cases of *M. tuberculosis* resistance to isoniazid in the clinical setting [9]. Resistance to isoniazid has also been associated with mutations in several other genes (*e.g. ndh*, *kasA* and *oxyR*–*ahpC* intergenic region) [10], but its direct association with resistance is still unclear.

Isoniazid is highly effective against *M. tuberculosis* (bactericidal at low concentrations), the reason why it remains a key component in multiple drug treatment regimens. However, resistant isolates are rapidly generated during monotherapy or inappropriate treatment, and many clinical isolates with no identified mutation have been described [9], [11]. As with other bacterial species, these resistant phenotypes also receive significant contributions from membrane transport proteins that prevent the compound from reaching the cellular target [12], [13]. The analysis of genome sequences has shown that mycobacteria have multiple putative efflux pumps [14] and to date, several pumps have been identified in various species of mycobacteria in association with low level resistance to various compounds, including isoniazid [15]–[20].

In general, increased activity of efflux systems is responsible for conferring low-level resistance to antibiotics, contrasting with the high-level resistance caused by mutations in genes encoding for the primary targets of these antibiotics [21]. Increased activity of efflux systems results in the reduction of intracellular levels of the antibiotic, which may enable the survival of a bacterial subpopulation under constant stress promoted by a sub-lethal level of antibiotic. During this period, mutants with alterations in the genes that favour resistance can be selected, therefore insuring the establishment of an antibiotic resistant population that is clinically significant [22]–[24]. It is this sub-population of bacteria that may then accumulate mutations with prolonged exposure to a constant concentration of antibiotic [25], [26].

Here, we investigated the mechanisms underlying the development of multidrug resistance in *M. tuberculosis* via the constant exposure of several isoniazid susceptible *M. tuberculosis* strains to the critical concentration of isoniazid, 0.1 µg/ml; followed by the evaluation of the effect of efflux inhibitors on the isoniazid minimum inhibitory concentration for the original and isoniazid exposed resistant strains. Analysis of gene expression of six efflux pumps related to isoniazid resistance in *M. tuberculosis* [12], [15], [19] and its correlation with the cell's ability to efflux ethidium bromide (a common efflux substrate), provides strong evidence that when challenged with isoniazid, *M. tuberculosis* reacts by a prompt efflux-mediated response. We further demonstrate that this isoniazid induced resistance can be reverted by efflux inhibitors, supporting their role as adjuvants in anti-tuberculosis therapy and prevention of MDR-TB emergence.

## Results

### Exposure to isoniazid

**Figure 1:**
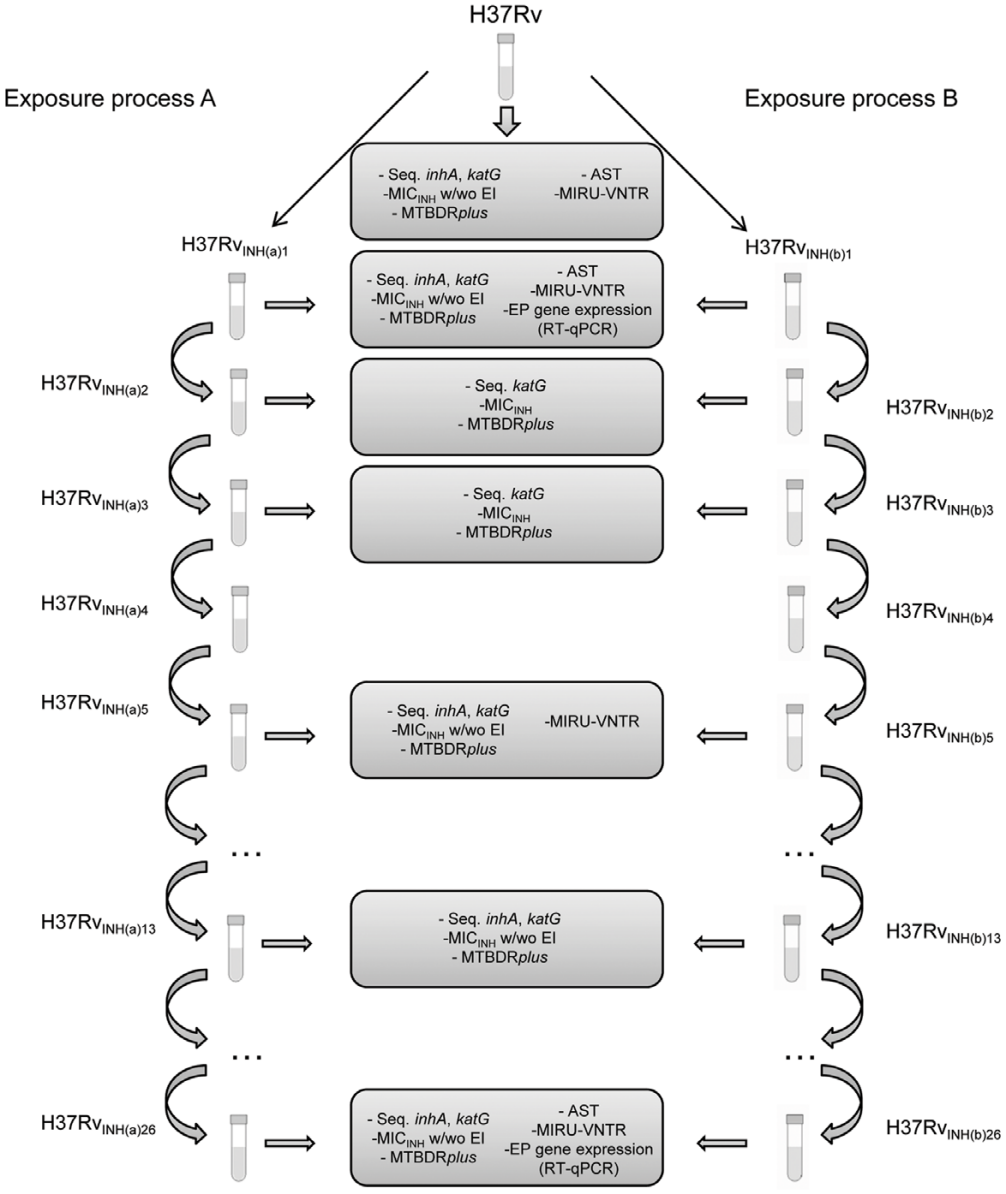
Schematic representation of exposure of strain H37Rv to 0.1 µg/ml INH using the BACTEC™ MGIT™ 960 and characterization assays performed at selected points. For each strain, exposure to INH was done in duplicate, in two independent assays - processes A and B. INH(a): exposure process A; INH(b): exposure process B; INH: isoniazid; EI: efflux inhibitor. Vertical arrows represent transfer to new MGIT tubes containing 0.1 µg/ml INH. Seq: nucleotide sequence determination for specific fragments of the genes involved in the resistance to INH; AST: susceptibility testing to all first line antibiotics. MIC_INH_: minimum inhibitory concentration determination of isoniazid. Note: This same procedure, here depicted as an example, was carried out for isoniazid exposure of each strain involved in this study.

Two *M. tuberculosis* strains susceptible to the first-line antibiotics (including the H37Rv reference strain) and two clinical strains monoresistant to rifampicin were constantly exposed to the critical concentration of isoniazid, 0.1 µg/ml, during an extended period of time – see Figure 1. Two independent exposure processes were carried out for each strain (exposure process A and B in Figure 1) to assess the stochastic behaviour of the biological events involved.

The effect of 0.1 µg/ml isoniazid exposure on the minimum inhibitory concentration of isoniazid (INH MIC) is summarised in Table 1. Briefly, INH MIC increases from an initial value of 0.05–0.1 µg/ml to as high as 256 µg/ml (Table 1). Exposure to isoniazid had no effect on the MIC of rifampicin against all strains (data not shown). Additionally, susceptibility testing for the five 1^st^ line antibiotics (streptomycin, isoniazid, rifampicin, ethambutol and pyrazinamide) applied to all strains at the beginning of the experiments and after the last passage in isoniazid indicated that development of resistance was restricted to isoniazid only (data not shown). We also observed that the time required for growth detection decreased with the number of passages (Table 1).

**Table 1:** The effect of serial passages in a constant concentration of isoniazid (0.1 µg/ml) on the INH MIC and the number of days required for detection of growth.

	INH MIC (µg/ml) (days required for detection of growth)						
	#0	#1	#2	#3	#5	#13	#26
**Fully susceptible strains**							
**H37Rv INH (a)**	0.05 (-)	**128 (19)**	128 (5)	128 (4)	128 (4)	128 (4)	**128 (4)**
**H37Rv INH (b)**	0.05 (-)	**128 (14)**	128 (8)	128 (5)	128 (3)	128 (3)	**128 (3)**
**401/06 INH (a)**	0.1 (-)	**256 (20)**	256 (6)	256 (4)	256 (4)	256 (4)	**256 (3)**
**401/06 INH (b)**	0.1 (-)	**64 (32)**	256 (14)	256 (18)	256 (7)	256 (4)	**256 (3)**
**Rifampicin monoresistant strains**							
**267/05 INH (a)**	0.1 (-)	**128 (15)**	128 (7)	128 (4)	128 (6)	128 (3)	**128 (3)**
**267/05 INH (b)**	0.1 (-)	**128 (17)**	128 (5)	128 (4)	128 (7)	128 (4)	**128 (3)**
**359/03 INH (a)**	0.05 (-)	**256 (18)**	256 (13)	256 (6)	256 (4)	256 (4)	**256 (3)**
**359/03 INH (b)**	0.05 (-)	**128 (17)**	256 (16)	256 (3)	256 (3)	256 (8)	**256 (3)**

**Legend:** INH: isoniazid; RIF: rifampicin; INH (a)/(b): adaptation processes to isoniazid A and B, respectively.

### Typing by MIRU-VNTR analysis

To confirm the isogenic nature of the exposed and initial parental strains, all strains were subjected to molecular typing by MIRU-VNTR analysis, which confirmed the identity of each culture during the antibiotic exposure processes (Tables 2 and 3).

**Table 2:** Genotypic characterization of the strains and derived cultures exposed to isoniazid (adaptation process A).

	Genotype MTBDR*plus*			DNA Sequencing		
Strain/Passage	*rpoB*	*mabA-inhA*	*katG*	*mabA-inhA*	*katG*	MIRU-VNTR profile
**H37Rv**	Wt	wt	wt	wt	wt	H37Rv
**H37Rv INH (a)1**	Wt	wt	wt	wt	wt	H37Rv
**H37Rv INH (a)2**	Wt	wt	wt	wt	wt	
**H37Rv INH (a)3**	Wt	wt	**Δ ** ***katG***	wt	**Δ ** ***katG***	
**H37Rv INH (a)5**	Wt	wt	**Δ ** ***katG***	wt	**Δ ** ***katG***	H37Rv
**H37Rv INH (a)13**	Wt	wt	**Δ ** ***katG***	wt	**Δ ** ***katG***	
**H37Rv INH (a)26**	Wt	wt	**Δ ** ***katG***	wt	**Δ ** ***katG***	H37Rv
**401/06**	Wt	wt	wt	wt	wt	A
**401/06 INH (a)5**	Wt	wt	wt	wt	wt	A
**401/06 INH (a)13**	Wt	wt	wt	wt	wt	
**401/06 INH (a)26**	Wt	wt	wt	wt	wt	A
**267/05**	S531L	wt	wt	wt	wt	B
**267/05 INH (a)5**	S531L	wt	wt	wt	wt	B
**267/05 INH (a)13**	S531L	wt	wt	wt	wt	
**267/05 INH (a)26**	S531L	wt	wt	wt	wt	B
**359/03**	S531L	wt	wt	wt	wt	C
**359/03 INH (a)1**	S531L	wt	wt	wt	wt	C
**359/03 INH (a)2**	S531L	wt	wt	wt	**TGG_438_→STOP**	
**359/03 INH (a)5**	S531L	wt	wt	wt	**TGG_438_→STOP**	C
**359/03 INH (a)13**	S531L	wt	wt	wt	**TGG_438_→STOP**	
**359/03 INH (a)26**	S531L	wt	wt	wt	**TGG_438_→STOP**	C

**Legend:** INH: isoniazid; RIF: rifampicin; wt: wild type; Δ: deletion of *katG* gene; S: serine; L: leucine. MIRU-VNTR profile A: 2,4,2,2,3,4,2,3,2,3,2,4,2,2,6,1,6,3,1,3,1,7,2,2; profile B: 2,4,4,2,3,4,3,3,2,4,2,4,2,2,6,1,5,3,1,3,1,5,2,2; profile C: 2,1,4,2,1,3,2,3,2,2,2,5,1,2,6,1,6,3,3,3,2,4,2,2.

**Table 3:** Genotypic characterization of the strains and derived cultures adapted to isoniazid (adaptation process B).

	Genotype MTBDR*plus*			DNA Sequencing		
Strain/Passage	*rpoB*	*mabA-inhA*	*katG*	*mabA-inhA*	*katG*	MIRU-VNTR profile
**H37Rv INH (b)5**	Wt	wt	wt	wt	wt	H37Rv
**H37Rv INH (b)13**	Wt	wt	wt	wt	wt	
**H37Rv INH (b)26**	Wt	wt	wt	wt	wt	H37Rv
**401/06 INH (b)5**	Wt	wt	wt	wt	wt	A
**401/06 INH (b)13**	Wt	wt	wt	wt	wt	
**401/06 INH (b)26**	Wt	wt	wt	wt	wt	A
**267/05 INH (b)5**	S531L	wt	wt	wt	wt	B
**267/05 INH (b)13**	S531L	wt	wt	wt	wt	
**267/05 INH (b)26**	S531L	wt	wt	wt	wt	B
**359/03 INH (b)5**	S531L	wt	wt	wt	wt	C
**359/03 INH (b)13**	S531L	wt	wt	wt	wt	
**359/03 INH (b)26**	S531L	wt	wt	wt	wt	C

Legend: INH: isoniazid; RIF: rifampicin; wt: wild type; S: serine; L: leucine. MIRU-VNTR profile A: 2,4,2,2,3,4,2,3,2,3,2,4,2,2,6,1,6,3,1,3,1,7,2,2; profile B: 2,4,4,2,3,4,3,3,2,4,2,4,2,2,6,1,5,3,1,3,1,5,2,2; profile C: 2,1,4,2,1,3,2,3,2,2,2,5,1,2,6,1,6,3,3,3,2,4,2,2.

### Detection of mutations associated with isoniazid resistance

Cultures corresponding to selected passages of isoniazid exposure were preliminarily monitored for mutations in *katG* and *mabA-inhA* operon by the Genotype MTBDR*plus* system. These strains were later analyzed by DNA sequencing of specific fragments of these same genes – see Tables 2 and 3.

**Figure 2:**
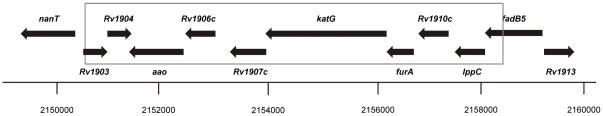
Map of the region deleted in the *M. tuberculosis* H37Rv reference strain as a result of the exposure to isoniazid. The region analyzed spans from positions 5′-2150314 to 5′-2159943 of the *M. tuberculosis* H37Rv genome sequence [14], adapted from Tuberculist, 2010, http://tuberculist.epfl.ch/. The area delimited corresponds to the fragment deleted in strain H37Rv_INH(a)3_.

For strain H37Rv, two different results were obtained for the two independent isoniazid exposure processes. In one of the duplicates, total deletion of *katG* gene was observed at passage #3 (H37Rv_INH(a)3_, GenBank accession number JQ406585). The precise extent and location of this deletion was defined by sequencing and chromosomal primer walking to be located between positions 5′-2150314 and 5′-2159943 of the *M. tuberculosis* H37Rv genome [14]. This deletion, 8084 bp in length, resulted in the complete loss of genes *Rv1903*, *Rv1904*, *aao*, *Rv1906c*, *Rv1907c*, *katG*, *furA*, *Rv1910c*, *lppc* and disruption of *fadB5* (Figure 2). Conversely, no alterations were detected on *katG* or any of the other gene targets tested during strain H37Rv second isoniazid exposure process (cf. Tables 2 and 3). Interestingly, both isoniazid-exposed cultures evidenced the same levels of resistance at equivalent passages of the exposure process (Table 1). For the three clinical strains subjected to the same isoniazid exposure process, no alterations were detected, except for the appearance of a STOP codon in position 1314 of the *katG* gene (codon 438) for strain 359/03 This alteration occurred at passage #2 (359/03_INH(a)2_, accession number JQ316462) of the first exposure process (Table 2), whereas no alteration was detected in the second exposure process of this same strain (Table 3). Again, no differences were observed between the isoniazid resistance levels of the two isoniazid-exposed 359/03 cultures (Table 1).

### Effect of EIs on the susceptibility to isoniazid

To test for the involvement of efflux on the increased resistance to isoniazid noticed through the exposure process, we determined the INH MIC in the first passage where this increased resistance was first noticed and in last passage (#26) of the exposure process, in the absence and presence of compounds known to act as efflux inhibitors (EIs). The compounds selected were thioridazine, chlorpromazine and verapamil, for which inhibitory activity against mycobacterial efflux pumps was already demonstrated [16], [27], [28]. The effects of these EIs on the INH MICs are summarized in Table 4. The INH MIC was reduced by thioridazine, chlorpromazine and verapamil to levels equal or below the critical concentration used for the standard susceptibility testing of this antibiotic in the majority of cases. We then assayed the efflux activity of these cultures by a semi-automated fluorometric method [27], [29], which uses the broad-range efflux substrate EtBr, in the presence and absence of an EI.

**Table 4:** MIC determination and susceptibility testing for the strains exposed to isoniazid in the presence and absence of efflux inhibitors.

	INH MIC (µg/ml) (Susceptibility testing for INH)										
	#0			#1				#26			
Strain	+TZ	+CPZ	+VP	no EI	+TZ	+CPZ	+VP	no EI	+TZ	+CPZ	+VP
**H37Rv INH (a)**	0.05 (S)	0.05 (S)	0.05 (S)	128 (R)	128 (R)	**0.05 (S)**	**0.06 (S)**	128 (R)	128 (R)	128 (R)	128 (R)
**H37Rv INH (b)**	0.05 (S)	0.05 (S)	0.05 (S)	128 (R)	128 (R)	**0.1 (S)**	**0.1 (S)**	128 (R)	128 (R)	**0.1 (S)**	**0.1 (S)**
**401/06 INH (a)**	0.1 (S)	0.1 (S)	0.1 (S)	256 (R)	256 (R)	128 (R)	256 (R)	256 (R)	256 (R)	128 (R)	256 (R)
**401/06 INH (b)**	0.1 (S)	0.1 (S)	0.1 (S)	64 (R)	**0.1(S)**	**0.1 (S)**	**0.1 (S)**	256 (R)	256 (R)	64 (R)	256 (R)
**267/05 INH (a)**	0.1 (S)	0.1 (S)	0.1 (S)	128 (R)	**0.1 (S)**	**0.1 (S)**	128 (R)	128 (R)	**0.1 (S)**	**0.1 (S)**	128 (R)
**267/05 INH (b)**	0.1 (S)	0.1 (S)	0.1 (S)	128 (R)	**0.1 (S)**	**0.1 (S)**	128 (R)	128 (R)	**0.1 (S)**	**0.1 (S)**	128 (R)
**359/03 INH (a)**	0.05 (S)	0.05 (S)	0.05 (S)	256 (R)	128 (R)	**0.1 (S)**	**0.03 (S)**	256 (R)	128 (R)	**0.1 (S)**	**0.03 (S)**
**359/03 INH (b)**	0.05 (S)	0.05 (S)	0.05 (S)	128 (R)	**0.1 (S)**	**0.1 (S)**	**0.1 (S)**	256 (R)	128 (R)	**0.1 (S)**	**0.1 (S)**

**Legend:** INH: isoniazid; INH (a): exposure process A; INH (b): exposure process B. S: susceptible; R: resistant. Values in bold correspond to full reversion of the INH resistance phenotype. EIs were used at ½ of their MIC. MICs for the EIs (passage 1): thioridazine (TZ): H37Rv and 359/03: 15 µg/ml, 401/06 and 267/05: 30 µg/ml; chlorpromazine (CPZ): 30 µg/ml for all strains; verapamil (VP): 256 µg/ml for all strains. MICs for the EIs (passage 26): TZ: H37Rv and 359/03: 15 µg/ml, 401/06(a)26: 15 µg/ml; 401/06(b)26: 30 µg/ml; 267/05: 30 µg/ml; CPZ: MIC of 30 µg/ml for all strains except H37Rv INH(a)26: 15 µg/ml; VP: 256 µg/ml for all strains except H37Rv INH(a)26 and 401/06: 128 µg/ml.

### Real-time detection of efflux activity

The assays were performed for all isoniazid non-exposed strains (#0) and at passages #1 and #26 of the two exposure processes (A and B).

**Figure 3:**
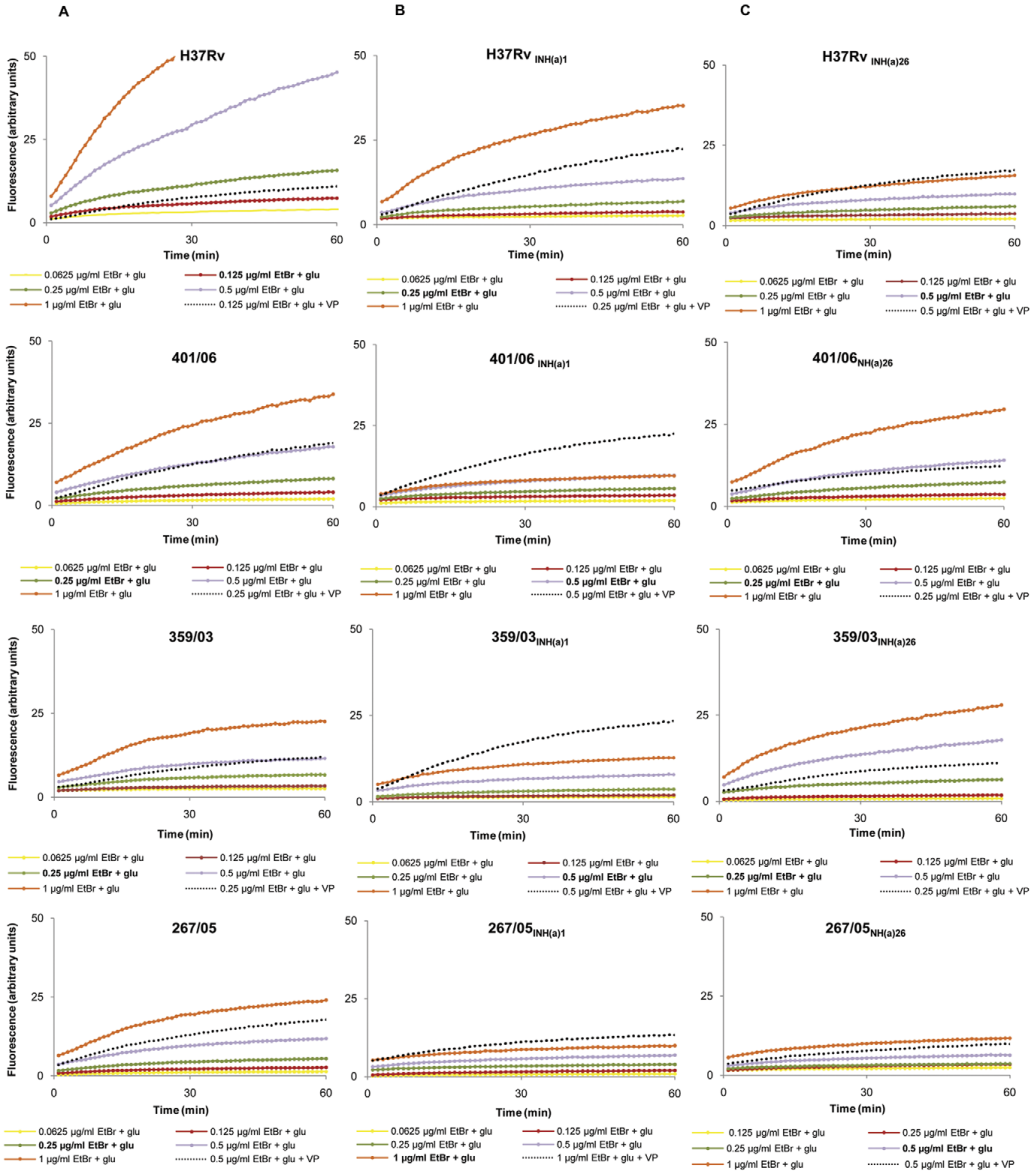
Accumulation of EtBr by the *M. tuberculosis* strains tested. The figure shows the accumulation of EtBr by the strains from adaptation process A as an example. The values at bold type correspond to the higher concentration of EtBr that cells can handle without detectable accumulation. The dotted line corresponds to the assay run using the EtBr concentrations for which influx-efflux are at equilibrium, in the presence of the EI verapamil, at sub-inhibitory concentrations. Panel (A): Parental strains (passage #0); Panel (B) strains after first passage with INH and Panel (C); strains after 26 passages with INH. INH: isoniazid.

The EtBr accumulation assays, used to determine the highest concentration of EtBr that cells can handle without detectable accumulation (see Material and Materials), showed that the clinical strains are able to handle higher EtBr concentrations than H37Rv (0.25–0.5 and 0.125 µg/ml of EtBr, respectively – see values at bold type in legends of Figure 3A). This means that the concentration at which EtBr influx and efflux reach steady state equilibrium is higher for the clinical strains than for H37Rv, *i.e*. that the former have higher efflux capacity than that of the reference strain [29]. With exposure to isoniazid, this efflux capacity increase, as shown by the flatness of the accumulation curves observed for all cultures at the first step of isoniazid exposure (Fig. 3B). With continuation of exposure to isoniazid, this efflux activity decreases, as shown by the lower EtBr concentrations needed to reach observable accumulation for cultures at passage #26 (cf. Fig. 3C with 3B), however not to the original levels shown by the non-exposed cultures (cf. Fig. 3C with 3A). Interestingly, this decrease of efflux activity with the prolonged exposure to isoniazid is observable for the clinical strains but not for H37Rv. For the sake of space, only the results for exposure process A are shown in Figure 3, but the same behavior was obtained for the exposure process B, for all the strains.

These assays were then performed using the EtBr concentrations for which influx-efflux are at equilibrium, but now in the presence of verapamil (efflux inhibitor) at sub-inhibitory concentrations (see dotted curves in Figure 3). Results clearly show that inhibition of efflux occurs, leading to an increased EtBr accumulation within cells. Again, this effect is more pronounced at the first step of isoniazid exposure (passage #1), except for H37Rv, which reinforces the hypothesis that this is the step in which the increase of efflux activity is more significant. Similar results were obtained for thioridazine and chlorpromazine, however with lower inhibitory effect (data not shown).

Finally, we evaluated the expression levels of genes coding for the main *M. tuberculosis* efflux pumps for cultures at the different isoniazid exposure steps.

### Expression analysis of genes coding for efflux pumps in response to isoniazid

The analysis of the relative expression of efflux pump genes previously identified as transporters of isoniazid was performed for all strains at passages #1 and #26. As detailed in Material and Methods, the expression levels of these genes were determined in the presence of isoniazid and compared to those obtained for the non-exposed culture (#0) [30].

Table 5 shows that the four strains reacted to the presence of isoniazid by overexpressing the majority of the efflux pump genes tested in a way that is not consistent among the two isoniazid exposures processes to which each strain was submitted. Additionally, and perhaps the most striking observation resulting from expression data, was the absence of a clearly defined pattern of gene expression in response to isoniazid exposure. Nevertheless, a general and strong increase in the expression was observed for the majority of the genes tested, *mmpL7*, *p55*, *efpA*, *mmr*, *Rv1258c* and *Rv2459.*

**Table 5:** Average quantification of the relative expression level, by RT-qPCR, of the genes that code for efflux pumps in *M. tuberculosis* in the two independent isoniazid exposure processes.

	Relative expression level ± SD					
	*mmpl7*	*Rv1258c*	*p55*	*efpA*	*mmr*	*Rv2459*
**Fully susceptible strains**						
**H37Rv INH(a)1**	**8.00±2.38**	**16.00±1.16**	**13.00±2.23**	**9.85±1.41**	**16.65±2.44**	**25.99±2.56**
**H37Rv INH(a)26**	0.44±0.12	1.99±0.66	0.82±0.15	0.34±0.28	0.44±0.16	0.50±0.02
**H37Rv INH(b)1**	**10.56±3.48**	**15.26±0.46**	**6.96±1.36**	**8.00±2.98**	**9.95±2.03**	**22.63±1.56**
**H37Rv INH(b)26**	**4.57±0.25**	2.53±0.03	2.70±0.43	**4.41±0.21**	1.54±0.29	2.31±0.13
**401/06 INH(a)1**	**34.30±1.24**	**22.63±0.47**	**18.38±1.94**	**16.00±1.32**	**24.25±2.34**	**9.19±0.11**
**401/06 INH(a)26**	**17.15±0.23**	**14.93±2.30**	**9.85±0.99**	**6.96±1.57**	**9.19±1.76**	**27.86±1.3**
**401/06 INH(b)1**	1.53±0.29	1.77±0.75	**3.47±1.16**	**6.50±1.30**	1.47±0.07	0.20±0.05
**401/06 INH(b)26**	**4.16±0.66**	**7.80±4.96**	**11.31±1.11**	**8.57±2.66**	2.29±0.77	2.64±0.39
**Rifampicin monoresistant strains**						
**267/05 INH(a)1**	**6.06±0.53**	**4.29±0.24**	**5.28±0.48**	**9.85±0.17**	3.73±0.13	**6.50±1.03**
**267/05 INH(a)26**	**115.56±59.44**	**53.85±2.64**	**110.80±66.17**	**341.05±66.43**	**59.06±53.66**	**32.00±1.98**
**267/05 INH(b)1**	**5.01±1.10**	2.00±0.21	3.10±0.43	**3.90±0.28**	2.62±0.54	1.69±0.36
**267/05 INH(b)26**	**17.15±1.24**	3.04±0.29	**12.13±0.67**	**22.63±3.09**	**3.89±0.57**	0.64±0.09
**359/03 INH(a)1**	**4.00±0.03**	1.62±0.54	2.08±1.06	1.87±0.00	2.03±0.86	0.76±0.09
**359/03 INH(a)26**	**13.96±1.36**	**9.01±2.17**	**8.53±2.86**	**10.66±2.07**	**11.46±4.9**	**32.00±2.01**
**359/03 INH(b)1**	**9.85±2.35**	**5.66±1.24**	**4.00±0.06**	**5.11±2.62**	**6.06±0.54**	0.25±0.03
**359/03 INH(b)26**	**25.99±1.33**	**29.86±3.29**	**29.86±2.56**	2.30±0.83	**10.56±1.36**	2.30±0.12

**Legend:** (a)1: exposition process A, first passage; (b)1: exposition process B, first passage; (a)26: exposition process A, passage twenty six; (b)26: exposition process B, passage twenty six. The relative expression of the efflux pump genes was assessed by comparison of the relative quantity of the respective mRNA in the presence of isoniazid to the non-exposed strain. Each culture was assayed in triplicate using total RNA obtained from three independent cultures. A level of relative expression equal to 1 indicates that the expression level was identical to the strain that was being compared. Genes showing expression levels equal or above four, when compared to the non-exposed strain, were considered to be overexpressed and are shown in bold.

## Discussion

In this work, we addressed three questions related to the molecular mechanisms involved in *M. tuberculosis* resistance to isoniazid: **i.** What are the effects of continuous exposure of *M. tuberculosis* strains to the critical concentration of isoniazid? **ii.** What are the biological events involved, in particular, which is the role played by efflux pumps in the emergence of resistance? and, **iii.** Does the final outcome vary when independent exposures are performed for the same strain?

For this purpose, we studied a panel of four *M. tuberculosis* strains, two fully susceptible and two monoresistant to rifampicin, through their independent exposure to a constant concentration of 0.1 µg/ml of isoniazid. During this process, strains became phenotypically resistant with an increase in their INH MIC exceeding 64 µg/ml, which is considered high-level resistance. The susceptibility towards the other 1^st^ line anti-tuberculosis drugs was not affected by this process, indicating the development of an isoniazid specific resistance mechanism.

In terms of the biological events occurring during exposure to isoniazid (question ii), we observed that for two strains, alterations occurred at the *katG* gene, corresponding to a full deletion in the case of H37Rv and the introduction of a stop codon for strain 359/03. Both alteration occurred early in the exposure process and were maintained for the remaining of the assays. These alterations in *katG* correlated with the loss of catalase activity (data not shown). The mutation found in clinical isolate 359/03 is rarely described in literature; however it fits into the known strategy of KatG functional weakening by introduction of mutations during isoniazid exposure [10], [31]. Others have also reported the *in vitro* deletion of total or part of the *katG* gene in strains exposed to isoniazid [31], [32], and total/partial deletion of the *katG* gene of isoniazid-resistant clinical isolates has also been previously reported [8], [33]. Interestingly, no genetic changes were detected in the second isoniazid-exposure procedure, run in parallel for these same strains, indicating that evolution of the same strain in the same conditions can bring about, in a non-deterministic course, cells that significantly differ both phenotypically and genetically.

Our results clearly show that, in addition to the occurrence of spontaneous mutations, efflux systems play a role in the development of isoniazid resistance. This occurs quite early during exposure to isoniazid and allows cells to survive in the presence of this antibiotic until a mutation conferring high level and stable resistance emerges. Recently, Srivastava *et al*., suggested a model for the development of drug resistance in the *M. tuberculosis* reference strain H37Rv, enabling the rapid emergence of high level resistance to both ethambutol and isoniazid [34]. In this model, it is proposed that induction of an efflux pump which transports two or more drugs is the first step to the emergence of resistance. Our results provide the experimental data that confirm the model proposed by these authors and demonstrate that this mechanism is extendable to clinical isolates.

Moreover, our work provides, for the first time, data captured on a real-time basis for increased efflux activity as the first-line response of *M. tuberculosis* to the critical concentration of isoniazid. This efflux-mediated response was detected for both susceptible and rifampicin monoresistant, reference or clinical strains and provide the cells with a rapid, non specific response to a highly noxious agent. As the isoniazid exposure process continues, two different patterns were observed: the susceptible reference strain H37Rv increased its efflux activity, even after deletion of the entire *katG* gene, whereas the clinical strains showed a decreased efflux activity in the last passage of isoniazid exposure. During the entire process, the clinical strains showed a capacity to handle higher EtBr concentrations than H37Rv, an additional evidence of their higher efflux capacity. Overall, the clinical strains appear to be more prompt to respond, via an efflux-mediated pathway, to noxious agents, such as EtBr or isoniazid, whereas H37Rv shows a less prompt, but more stable/prolonged use of efflux as a detoxifying response to these drugs. These results suggest that clinical *M. tuberculosis* strains are primed to efflux noxious compounds, as already observed in other bacteria [35]. The presence of such efflux system(s) and their role in resistance to these drugs was additionally confirmed by the use of efflux inhibitors in both real-time efflux assays and MIC determinations. While in the first assays, these compounds were able to reduce efflux of EtBr, their use in INH MIC determination showed the involvement of efflux on the high level resistance to this antibiotic. For some strains, the reduction on INH MIC by the EIs tested reached levels identical to their susceptible parental counterparts. This clearly shows that in these cases, high level isoniazid resistance is mainly efflux-driven. From the several inhibitors tested, chlorpromazine and verapamil were the two most effective for inhibiting isoniazid efflux in *M. tuberculosis*, as previously demonstrated for *M. tuberculosis* complex [36].

It is worth noticing that EIs show a more significant effect on the MICs for cultures at first passage compared to their effect on the MICs of the last passage (#26). For strain 401/06, submitted to exposure process A, despite the fact that no evident genetic alteration was detected, the high INH MIC obtained after isoniazid exposure could not be reduced by any of the EIs tested (Table 4). This result suggests that, for this culture, the isoniazid high level resistance detectable at first passage is already mutation-driven. Interestingly strain 267/05, without detectable mutations is the one with the highest level of expression of genes for efflux further supporting that overexpression of efflux-pumps can sustain isoniazid resistance to levels as high as those achieved by the canonical mutations.

To identify the efflux system(s) involved in this first-line response to isoniazid, we selected a set of genes coding for efflux pumps reported to be involved in the transport of noxious substances, including isoniazid [12], [15], [37]. The detection by RT-qPCR of highly increased expression of these genes following isoniazid exposure, further evidences that an efflux-mediated response provides an early stress response that creates opportunity for other resistance mechanisms to arise.

Although we detected a general and marked increase of efflux pumps genes, most of which being significantly overexpressed, we also noted the absence of a clearly defined pattern of specific gene expression in response to isoniazid exposure. Efflux pumps seem to be promiscuous in their activity as we cannot associate extrusion of isoniazid to a specific gene. Similar results were obtained by others regarding the extrusion of rifampicin [38]. As described in Materials and Methods, the RT-qPCR data were analyzed considering a cut-off value of fourfold as corresponding to significant overexpression [39]. This can be considered a stringent cut-off and somehow limit our analysis [40], since the levels of gene expression obtained, in comparison to the non-exposed condition, lied in the majority of the cases, above two/three fold. Nevertheless, even considering the more stringent value of four, a clear and general ability to trigger efflux pump genes overexpression in response to isoniazid presence was observed along the exposure processes, for all strains. The genes for which a more consistent isoniazid-mediated response was observed, were the genes involved in the transport and synthesis of mycolic acids, *mmpL7* and *efpA* respectively [41], [42], and *p55*, considered to be involved in isoniazid transport [17], [20], [38], [43], [44]. Again, our study complements other earlier findings [15], [34], [36], who suggested the involvement of these genes in the resistance to isoniazid, by providing experimental data showing that susceptible reference strain and clinical strains use these pumps as an immediate response to the presence of isoniazid concentrations that are considered to be inhibitory.

Finally, concerning the third question raised in this work - does the final outcome vary when independent exposures are performed for the same strain, we have found that each strain may differ at the final outcome of the process of its exposure to the isoniazid critical concentration, in terms of the resistance mechanism it may adopt (mutations in different target genes, etc), although no differences were observed at the resistance level, which was always well above 64 µg/ml of isoniazid. Nevertheless, they all respond in a similar way at the first steps of this process and that is through isoniazid efflux, which may constitute an early stress response of bacteria against environmental noxious agents such as appears to be the case for isoniazid. After this first, efflux-mediated response, evolution may take different non-deterministic paths conducting to high level resistance. Collectively, these observations support the experimental strategy followed in this work that highlighted alternative pathways by which the same *M. tuberculosis* strain responds to 0.1 µg/ml isoniazid, all resulting in the same high resistance level.

In conclusion, constant exposure of *M. tuberculosis* to the commonly used critical concentration of isoniazid causes susceptible strains to become highly resistant to this key anti-tuberculosis drug. The same procedure applied to strains initially monoresistant to rifampin results in the development of multidrug resistance as defined by the WHO, *i.e*, resistance to isoniazid and rifampicin. To our knowledge, this is the first presentation of an *in vitro* process that mimics the development of multidrug resistant *M. tuberculosis* strains, which correlates with the anticipated development of MDR-TB in a patient treated for prolonged periods with a constant dose of isoniazid, as needed for effective therapy. Therefore, the results obtained in this work emphasize the need for revising isoniazid critical concentration and reinforce the importance of multiple drug therapy in all anti-tuberculosis regimens [45], [46]. Furthermore, efflux inhibitors like the ones tested in this work represent relevant alternatives in the search for new effective compounds and new therapeutic strategies for preventing the emergence of and possibly in the treatment of MDR-TB.

## Materials and Methods

### *M. tuberculosis* strains

The strains studied included two *M. tuberculosis* strains susceptible to the first-line antibiotics, the reference strain H37Rv ATCC27294^T^ and a clinical isolate 401/06, plus; two clinical isolates 359/03 and 267/05, both monoresistant to rifampicin, harboring the most common *rpoB* mutation in clinical isolates, S531L, all from the culture collection of Grupo de Micobactérias, Unidade de Microbiologia Médica, Instituto de Higiene e Medicina Tropical (IHMT, UNL).

Cultures, susceptibility testing, minimum inhibitory concentration (MIC) determination and antibiotic exposure process was conducted using the BACTEC™ MGIT™ 960 system (BACTEC 960) and the Epicenter V5.53A software equipped with the TB eXIST module (Becton Dickinson Diagnostic Systems, Sparks, MD, USA).

### Antimicrobial agents

The lyophilized drugs (BACTEC™ MGIT™ 960 SIRE and PZA kits; SIRE: streptomycin, isoniazid, rifampicin and ethambutol; PZA: pyrazinamide) used in the standard susceptibility testing and in the exposure process to isoniazid and rifampicin were purchased from Becton Dickinson and the stock solutions prepared as per the manufacturer's instructions. Isoniazid for MIC determination and efflux inhibitors verapamil, thioridazine and chlorpromazine, as well as the efflux substrate ethidium bromide (EtBr), were purchased from Sigma-Aldrich (St. Louis, MO, USA). All drugs were prepared in sterile deionized water.

### Exposure process to the critical concentration of isoniazid

Each strain was exposed to isoniazid (0.1 µg/ml) in duplicate (Figure 1 – Schematic example for isoniazid exposure of strain H37Rv ATCC27294^T^). This concentration is defined as the lowest concentration necessary to inhibit 99% of the wild-type strains of *M. tuberculosis* that were never in contact with this antibiotic, and is the critical concentration used for the BACTEC™ MGIT™ 960 SIRE AST procedure [47], [48]. Briefly, the exposure process for strains susceptible to isoniazid began with the preparation of MGIT tubes containing SIRE supplement (Becton Dickinson) and 0.1 µg/ml of isoniazid. These tubes were then inoculated with 0.5 ml of the initial culture and subsequently incubated at 37°C in the BACTEC 960 system until full growth was evident. For each strain, this process was done in duplicate – see Figure 1 for example.

For convenience, the various passages of the strains are identified as follows: strain number, antibiotic, exposure process (A or B) and the number of the passage. For example, H37Rv_INH (a)26_ refers to strain H37Rv exposed to isoniazid, exposure process A, passage 26 – see Figure 1 for example.

### MIC determination and antibiotic susceptibility testing

#### (i) MIC determination

The MICs of the antibiotics and efflux inhibitors (EIs) were performed in accordance to the procedures issued by the manufacturer of the BACTEC 960 system revised by Springer *et al*. [48], [49] for the parental strains at the initial process and periodically for each of the progeny cultures (Figure 1). The concentrations used were as follows: isoniazid: 0.025 to 256 µg/ml; verapamil: 30 to 512 µg/ml; thioridazine and chlorpromazine: 7.5 to 60 µg/ml; EtBr: 0.25 to 4 µg/ml. At the time of testing, two-fold serial dilutions were prepared to achieve the desired concentrations. Each drug-containing tube was inoculated with 0.8 ml of SIRE supplement, 0.1 ml of each drug in the appropriated concentration and 0.5 ml of the culture. For the preparation of the drug-free growth control tube (proportional control), the culture was diluted 1∶100 with a sterile saline solution and 0.5 ml transferred into a new MGIT tube. Additionally, a second drug-free growth control, inoculated with 0.5 ml of the undiluted suspension of the strain, was prepared and served as absolute control for inoculums errors. The tubes were inserted in the BACTEC 960 system and growth monitored with the TB eXIST module. The interpretation of the results was performed as proposed by Springer *et al*. [49].

#### (ii) Isoniazid MIC determination in the presence of EIs

The MICs of isoniazid (INH MIC) in combination with the EIs were performed in the first passage where the increased resistance was first noticed and in the last passage (#26) of the serial exposure process to isoniazid – Figure 1. The EIs were used at a concentration corresponding to ½ of the respective MIC. This concentration was selected since it has no effect on the growth of the strains following the protocol described above.

#### (iii) Susceptibility testing in the presence and absence of EIs

For standard susceptibility testing against isoniazid, rifampicin, pyrazinamide, streptomycin and ethambutol, the readings were automatically interpreted by the BACTEC 960 system and reported as either susceptible or resistant. The preparation of the drug containing tubes and controls was done as described above. For the susceptibility testing for isoniazid in the presence of the EIs, the tubes containing 0.1 µg/ml of isoniazid were inoculated with the EI at ½ of the MIC.

### Genotypic characterization of the strains

#### (i) DNA extraction

Genomic DNA was extracted using the QIAamp DNA mini kit (QIAGEN, GmbH, Hilden, Germany) according to the manufacturer's instructions.

#### (ii) Screening of mutations

The most common mutations in *rpoB*, *katG* and the *mabA-inhA* operon were screened during the exposure process, using the system Genotype MTBDR*plus* (Hain Lifescience GmbH, Nehren, Germany) according to the manufacturer's instructions.

#### (iii) DNA sequencing

The analysis of internal fragments of the genes associated with isoniazid resistance, *katG* and the *mabA-inhA* operon, was performed according to Perdigão *et al*. [50].

#### (iv) MIRU-VNTR analysis

MIRU-VNTR genotyping was performed for each strain and at defined passages of the exposure process by multiplex PCR amplification of 24 MIRU–VNTR loci, as described by Supply *et al*. [51].

### Quantification of expression of genes coding for efflux pumps by RT-qPCR

#### (i) RNA extraction

Total RNA was isolated from the cells using the RNeasy mini kit (QIAGEN) according to the manufacturer's instructions. Briefly, from a culture with 100–200 GU (about 10^6^–10^8^ cells/ml), 1 ml aliquot was removed and centrifuged at 13 000 rpm during 10 minutes. Then, 500 µl supernatant was removed and 1 ml of RNA*protect Bacteria reagent* (QIAGEN) added. An enzymatic lysis step was carried out with lysozyme at 3 µg/ml (Sigma) for 10 minutes, followed by lysis in an ultrasonic bath at 35 kHz (Gen-Probe, California, USA) during 15 minutes. The RNA was then purified using the RNeasy kit (QIAGEN) and treated with RNase-free DNase I (QIAGEN) during 2 hours and 15 minutes by on-column digestion at room temperature to reduce the presence of contaminating DNA. All RNA samples were aliquoted and frozen at -20°C until required.

#### (ii) RT-qPCR assay

The relative expression level of the genes that code for the main membrane efflux transporters in *M. tuberculosis* (*mmpL7*, *p55*, *efpA*, *mmr*, *Rv1258c* and *Rv2459*) were analyzed by RT-qPCR in the first passage where the increased resistance was first noticed and in last passage (#26) of the exposure process to isoniazid – Figure 1. The normalization of the data was done using the *M. tuberculosis* 16S rDNA for each experiment. The forward and reversed primers employed are described in Table 6. The RT-qPCR procedure was performed in a Rotor-Gene™ 3000 thermocycler and followed the protocol recommended for use with the QuantiTect SYBR Green RT-PCR Kit (QIAGEN). The determination of the relative mRNA expression level was performed using the comparative quantification cycle (*Cq*) method [40]. The relative expression of the six efflux pump genes analyzed was assessed by comparison of the relative quantity of the respective mRNA in the presence of isoniazid to the non-exposed culture, following the same technical approach previously published [30]. Each culture was assayed in triplicate using total RNA obtained from three independent cultures. A level of relative expression equal to 1 indicates that the expression level was identical to the unexposed strain. Genes showing expression levels equal or above four, when compared with the unexposed strain, were considered to be overexpressed [39].

**Table 6:** Sequences of the primers used in the RT-qPCR assays.

Gene	Primer Sequence (5′-3′)	Amplification product (bp)	Reference
*mmpL7_*Fw	TAC CCA AGC TGG AAA CAA	214	[36]
*mmpL7*_Rv	CCG TCA GAA TAG AGG AAC CAG	214	[36]
*p55_*Fw	AGT GGG AAA TAA GCC AGT AA	198	[36]
*p55_*Rv	TGG TTG ATG TCG AGC TGT	198	[36]
*efpA_*Fw	ATG GTA ATG CCT GAC ATC C	131	[36]
*efpA_*Rv	CTA CGG GAA ACC AAC AAA G	131	[36]
*mmr_*Fw	AAC CAG CCT GCT CAA AAG	221	[36]
*mmr_*Rv	CAA CCA CCT TCA TCA CAG A	221	[36]
*Rv1258c_Fw*	AGT TAT AGA TCG GCT GGA TG	268	[36]
*Rv1258c_Rv*	GTG CTG TTC CCG AAA TAC	268	[36]
*Rv2459_Fw*	CAT CTT CAT GGT GTT CGT G	232	This study
*Rv2459_Rv*	CGG TAG CAC ACA GAC AAT AG	232	This study
16S*_*Fw	CAA GGC TAA AAC TCA AAG GA	197	[36]
16S*_*Rv	GGA CTT AAC CCA ACA TCT CA	197	[36]

FW: forward; RV: reverse.

### Semi-automated fluorometric method

This method allows the real-time fluorometric detection of the accumulation and extrusion of EtBr, using the Rotor-Gene 3000™ thermocycler (Corbett Research, Sidney, Australia) [29], [52]. The assays were performed based on the protocol previously described [28], [29], [52] with modifications due to the growth features of this microorganism, mainly the slow generation time and the minimization of cell clumps. Increased biosafety measures were taken to prevent the production and dispersal of aerosols with infective particles since we were dealing with a Level 3 pathogen. The semi-automated fluorometric method was applied to the initial strains and to the isoniazid exposed strains, at the first passage where the increased resistance was first noticed and in the last passage (#26) of the adaptation process to isoniazid independently of the genetic background of each adapted culture. The strains were grown in 100 ml of Middlebrook 7H9 medium (DIFCO, Madrid, Spain) in Erlenmeyer flasks containing 10% OADC enrichment (Becton Dickinson) and 0.05% Tween 80. All cultures were incubated at 37°C, without stirring, until they reached an approximate optical density at 600 nm (OD_600_) of 0.8 (mid-log phase). After the cultures reached the desired OD_600_, 25 ml cultures were centrifuged at 2700 g during 3 minutes at 25°C. After this, the supernatant were discarded, the pellet washed, resuspended in PBS and centrifuged as before. This procedure was performed twice. For accumulation assays, the washed cells were re-suspended in PBS and the OD_600_ adjusted to 0.8. In order to determine the lowest concentration of EtBr that causes accumulation, 50 µl of the bacterial suspension was added to 0.2 ml PCR tubes containing different concentrations of EtBr that ranged from 0.0625 to 5 µg/ml and glucose at a final concentration of 0.4%. The final OD_600_ of the bacterial suspension in the assay was 0.4. The assays were conducted at 37°C in a Rotor-Gene 3000™, and the fluorescence of EtBr was measured (530/585 nm) at the end of each cycle of 60 seconds, for 60 minutes. After determining the higher concentration of EtBr that do not causes accumulation, the effect of the EIs verapamil, thioridazine and chlorpromazine on the accumulation of EtBr was evaluated. These assays were performed like described above with each EI at ½ of the MIC, EtBr at the higher concentration that do not cause accumulation (determined for each strain and adapted cultures), 37°C and with glucose.
